# Aggressive Locoregional Treatment Improves the Outcome of Liver Metastases from Grade 3 Gastroenteropancreatic Neuroendocrine Tumors

**DOI:** 10.1097/MD.0000000000001429

**Published:** 2015-08-28

**Authors:** Shunda Du, Jianjiao Ni, Linqian Weng, Fei Ma, Shaohua Li, Wenze Wang, Xinting Sang, Xin Lu, Shouxian Zhong, Yilei Mao

**Affiliations:** From the Department of Liver Surgery (SD, JN, LW, XS, XL, SZ, YM); Department of Medical Oncology, Cancer Hospital and Institute, Chinese Academy of Medical Sciences and PUMC, Beijing (FM); Department of Hepatobiliary Surgery, Sun Yat-sen University Cancer Center, Guangzhou (SL); and Department of Pathology, Peking Union Medical College (PUMC) Hospital, Chinese Academy of Medical Sciences and PUMC, Beijing, China (WW).

## Abstract

Grade 3 (G3) gastroenteropancreatic (GEP) neuroendocrine tumors (NETs) are rare, and there is no report specifically dealing with patients of liver metastases from G3 GEP NETs.

From January 2004 to January 2014, 36 conservative patients with G3 GEP NET liver metastases were retrospectively identified from 3 hepatobiliary centers in China. The clinical features and treatment outcomes were analyzed.

Aggressive locoregional treatments (LT, including cytoreductive surgery, radiofrequency ablation, and liver-directed intra-arterial intervention) and systemic therapy (ST) were introduced separately or combined, with 26 (72%) patients receiving resection of primary tumor and/or hepatic metastases, 12 patients receiving non-surgical locoregional interventions (NSLRIs), and 22 patients receiving certain kind of STs. Median overall survival (OS) was 20.0 months (95% confidence interval [CI]: 8.9–31.1 months) and survival rates were 62.6%, 30.1%, and 19.8%, at 1, 3, and 5 years, respectively. The median OS was 9.0 months (95%CI: 3.3–14.7 months) for patients receiving only STs (n = 6), 19 months (95%CI: 1.3–36.8 months) for patients receiving LT followed by STs (n = 16), and 101 months (95%CI: 0.0–210.2 months) for patients receiving only LT (n = 12). Moreover, compared with those receiving only ST or best supportive care, patients given certain types of LTs had higher rates of symptom alleviation (3/8 versus 20/23). On univariate analysis, positive prognostic factors of survival were pancreatic primary tumor (*P* = 0.013), normal total bilirubin level (*P* = 0.035), receiving surgery (*P* = 0.034), receiving NSLRI (*P* = 0.014), and sum of diameters of remnant tumor < 5 cm (*P* = 0.008). On multivariate analyses, pancreatic primary tumor (*P* = 0.015), normal total bilirubin level (*P* = 0.002), and sum of diameters of remnant tumor < 5 cm (*P* = 0.001) remained to be independent prognostic factors.

For patients with G3 GEP NET liver metastases, aggressive LTs may improve clinical outcomes. Larger studies with prospective design are warranted to consolidate these results, and to discover the most appropriate seletion criteria for patients to undergo different kinds of aggressive LTs and to find the most effective combinations, with or without ST.

## INTRODUCTION

Gastroenteropancreatic (GEP) neuroendocrine tumors (NETs) are a heterogeneous group of malignancies.^1^ They were previously regarded as rare, but in fact are increasing in incidence^2^ (3.65 per 100,000 individuals per year).^3^ Based on the 2010 World Health Organization (WHO) classification, NETs can be divided into 3 groups: Grade 1 (Ki-67 ≤2%), Grade 2 (Ki-67 3%–20%), and Grade 3 (G3, Ki-67 > 20%).^4^ G3 NETs are also called neuroendocrine carcinomas. The GEP tract is the most common site for extrapulmonary neuroendocrine carcinomas and accounts for 35% to 55% of all cases.^5^ About 40% to 95% GEP NETs are metastatic at diagnosis^6^ and liver metastases are observed in 28.3% to 77% of patients with pancreatic NETs, and 67% to 91% of patients with small bowel NETs.^7^

A combination of systemic platinum-based chemotherapy with local treatment consisting of radiotherapy and/or surgery offers the best chance for long-term survival in patients with limited G3 NETs.^5,6^ However, liver metastases from G3 NETs are generally considered not amenable for resection (with multifocal or bilobar growth, or both, and anticipated high recurrence rates) and systemic therapy (ST) are recommended to be the first-line choice,^6,7^ which has not been fully justified in large randomized studies.

Our previous study,^8^ examining the largest dataset from Asia, demonstrated that surgical resection improved patient outcome irrespective of the pathological grade of the tumor. However, patients with different grades were mixed and the sample size of G3 patients was quite small.

### PATIENTS AND METHODS

We reviewed the clinical records of patients with histologically confirmed diagnosis of G3 GEP NETs made between 2004 and 2014 at 3 major hepatobiliary centers in China (Peking Union Medical College Hospital, Beijing; Cancer Institute & Hospital, Chinese Academy of Medical Sciences, Beijing; and Sun Yat-Sen University Cancer Center, Guangzhou). The study protocol was approved by the Ethical Committee of the 3 hospitals.

### Data Collection

Standard demographic and clinicopathologic data were collected from each patient, including demographics, symptoms, presence of concomitant extrahepatic metastatic disease, presentation relative to the primary tumor (synchronous vs metachronous), primary and metastatic tumor characteristics, liver function tests and radiological images, treatment and complications, most recent follow-up date, vital status (alive vs dead), and date of death.

To clarify the relationship between tumor burden and disease prognosis, we calculated sum of the diameters of primary tumors and metastatic lesions from radiological images such as computed tomography, MRI, ultrasound, and positron emission tomography-computed tomography, at presentation of the disease and at the first follow-up after the major treatment.

### Statistical Analyses

Overall survival (OS) was defined as the time from identification of liver metastasis to the date of last follow-up or death. Cumulative event rates were calculated and survival was estimated using the Kaplan–Meier method. Univariate analyses were performed using the log-rank test to compare differences between categorical groups. Relative risk is expressed as hazard ratios (HR) with 95% confidence interval (CI). Independent prognostic factors were determined using a multivariable Cox proportional hazards regression model with a forward stepwise Wald selection method. Significance levels were set at *P* = 0.05. All tests were 2-sided. All statistical analyses were performed using SPSS version 12.0 (SPSS Inc, Chicago, IL).

## RESULTS

### Patient and Tumor Characteristics

Table 1 presents the clinicopathologic features of the 36 patients in this study. Overall, most of the patients had symptoms attributable to mass effect or hormonal hypersecretion, with abdominal pain being the most common presenting manifestation (n = 23, 63.9%). In addition, hematochezia, diarrhea, flush, jaundice, vomiting, and fever were also found. In terms of primary tumor location, a third of cases originated from the pancreas, and the others were from the digestive tract, including the stomach (n = 7), small bowel (n = 5), colon (n = 4), esophagus (n = 3), gall bladder (n = 3), and rectum (n = 2). Primary lesions were solitary in all patients, with a median size of 44 mm. Metachronous hepatic metastases were present in 11 cases, with a median interval between the diagnosis of primary tumor and the presentation of hepatic lesions of 7 months (range, 1–53 months).

**TABLE 1:**
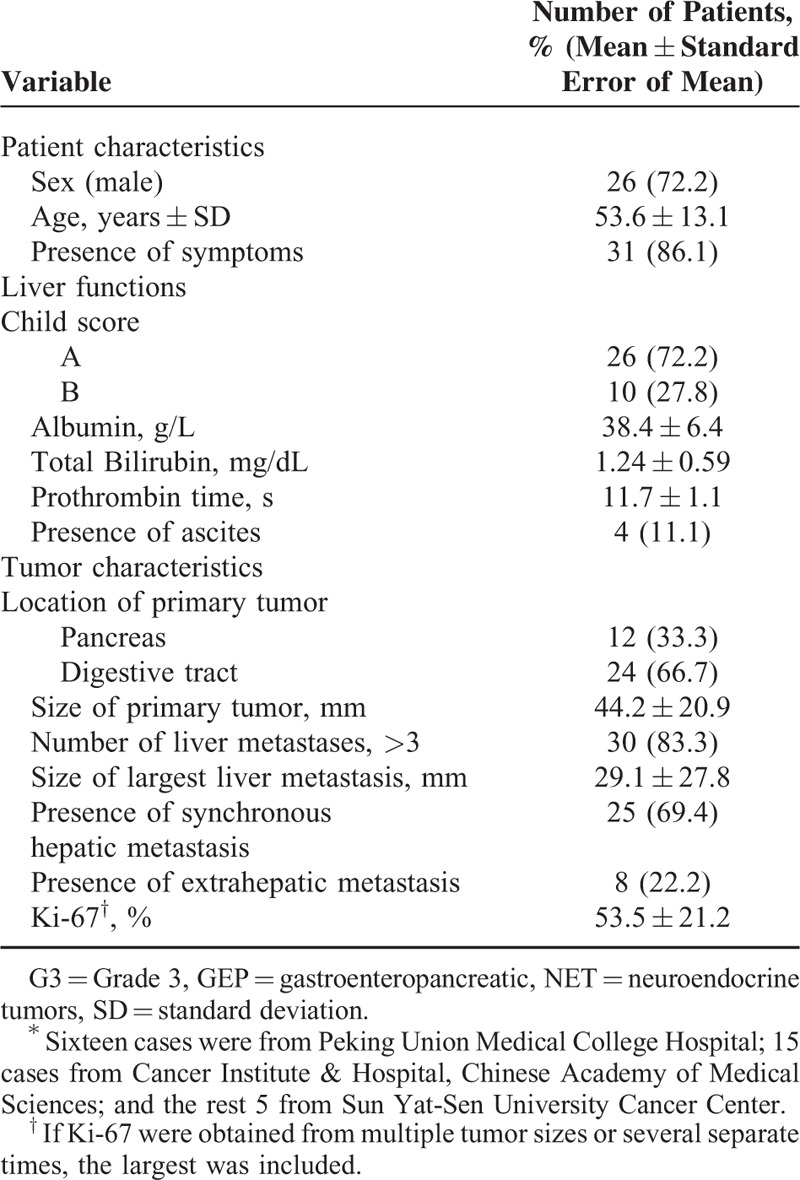
Demographic and Clinical Characteristics of Patients With GEP G3 NET Liver Metastases^∗^

The majority of hepatic metastases were diffuse with bilobar distribution and a median size of the largest lesion of 29 mm. Extrahepatic metastases were discovered in 8 patients, with lung being the most common site of extrahepatic lesions (n = 4). As for liver function at the baseline, albumin decreased in 10 cases (range, 28–35 g/L). Total bilirubin increased in 8 cases (range, 1.12–16.6 mg/dL). Ascites was found in 4 cases and prothrombin time was within normal limits in all cases. In addition, two thirds of the patients were categorized as Child–Pugh Score A.

#### Treatment Modalities and Safety

Cytoreductive surgeries were performed in 26 cases. Resection of the primary tumor only was carried out in 16 patients with unresectable liver metastases, including esophagectomy, gastrectomy, pancreatectomy with or without splenectomy, cholecystectomy, and coloproctectomy. Hepatic surgeries were done in 5 patients with primary tumors not amenable for resection, including both anatomic resection and nonanatomic tumor excision. Resection of both primary and metastatic lesions were implemented in the remaining 5 patients, most of which were performed simultaneously. No liver transplantation was performed.

The operations were generally well tolerated, with only 2 patients suffering from complications. A 61-year-old man receiving distal pancreatectomy with splenectomy, developed pancreatic fistula. After symptomatic treatment, the patient gradually recovered and was still alive 31 months after the operation at the last follow-up. Another case was a 53-year-old woman, cholecystectomy and left hemihepatectomy was done. She developed hepatic failure and died 7 months after the operation, with the exact cause of death undetermined.

In addition, 3 patients received radiofrequency ablations, and the number of procedures ranged from 1 to 3. Nine patients adopted liver-directed intra-arterial interventions, 8 of which were transarterial chemoembolization, and the number of procedures ranged from 1 to 9. No significant complications related to these non-surgical locoregional interventions (NSLRIs) were observed.

STs were administered as adjuvant treatment for more than half of the patients and were introduced to patients with unresectable disease or those refusing aggressive treatment. Platinum-based cytotoxic chemotherapies were used in 21 patients, and long-term octreotide and interferons were administered in 2 patients, respectively. No unexpected side effects were observed. Two patients with heavy tumor burden and sever comorbidities received only best supportive care.

#### Aggressive Locoregional Treatment Improves Outcome

With a median follow-up of 36 months (range, 2–101 months), 23 patients died and median OS was 20.0 months (95%CI: 8.9–31.1 months), with a survival rates of 62.6%, 30.1%, 19.8%, at 1, 3, and 5 years, respectively.

**FIGURE 1:**
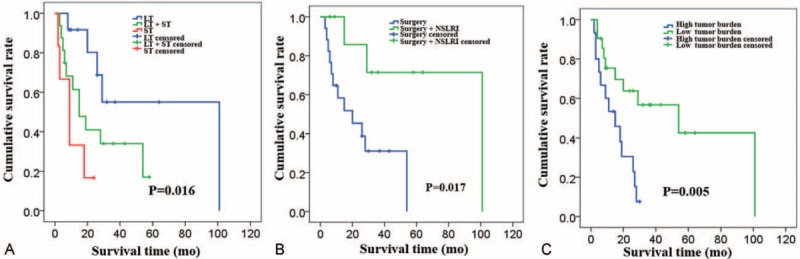
Kaplan–Meier overall survival stratified by treatment strategies and prognostic factors. (A) Median OS for patients stratified by receiving only LT versus only ST, versus LT followed by ST (patients receiving only best supportive care were excluded). (B) Median OS for patients stratified by receiving only surgery versus surgery + NSLRI (patients that did not receive surgery were excluded). (C) Median OS for patients stratified by remnant tumor burden and sum of diameters >5 cm was considered “high tumor burden,” while those ≤5 cm was considered “low tumor burden.” LT = locoregional treatment, NSLRI =  non-surgical locoregional interventions, OS = overall survival, ST = systemic therapy.

First, surgery and NSLRI prolonged patient survival, and the difference reached statistical significance (Table 2). The median OS and survival rate in the surgery group were significantly better than the non-surgery group, which applied to the comparison between NSLRI and non-NSLRI group. To further test this conclusion, we stratified patients according to the treatment strategies and performed survival tests using Kaplan–Meier models. For the purposes of analyses, surgery, ablation, and liver-directed intra-arterial interventions were combined and categorized as “locoregional treatment (LT).” Compared with ST, LT significantly improved survival, as median OS was 9 months (95%CI: 3.3–14.7 months) in patients receiving only ST, 101 months (95%CI: 0.0–210.2 months) in patients receiving only LT, 19 months (95%CI: 1.3–36.8 months) in patients receiving LT followed by ST (*P* = 0.016; Figure 1A).

**TABLE 2:**
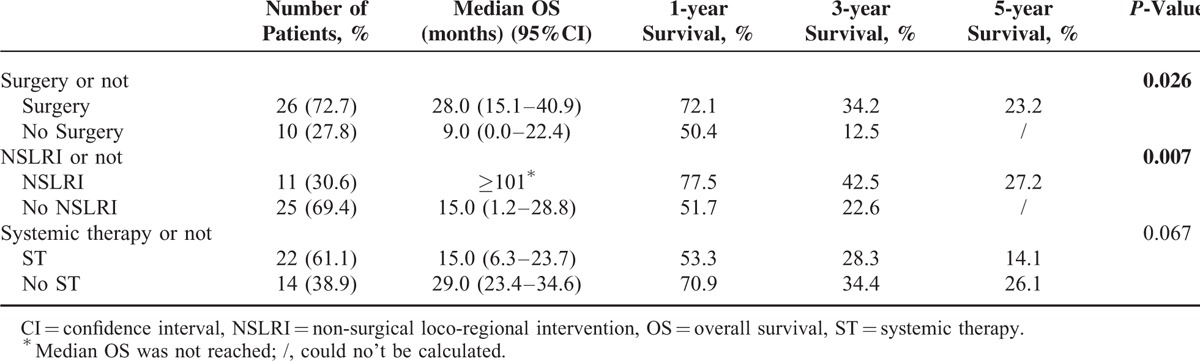
Intervention-Related Subgroup Analysis

Second, the combination of surgery and NSLRI seemed to offer the longest overall survival. Median OS of patients receiving only NSLRI was 19 months, which is comparable to that of patients receiving only surgery (20 months, 95%CI: 1.2–38.8 months). Median OS of patients receiving both surgery and NSLRI exceeded 101 months, which was significantly longer (*P* = 0.017; Figure 1B).

In addition, aggressive LT contributed to alleviating symptoms and improving quality of life. In total, 20 of the 23 symptomatic patients who received aggressive LT experienced complete (n = 7) or partial (n = 16) alleviation of tumor-associated symptoms, and this percentage was much higher than in the conservative treatment group (3 of 8 symptomatic patients in this group experienced partial alleviation and no complete alleviation was observed).

#### Primary Tumor Site, Liver Function, Tumor Burden, and Survival

On univariate analyses, clinicopathologic factors known to be associated with prognosis of G3 NETs or neuroendocrine liver metastasis (NELMs) were analyzed to determine their association with OS. Factors influencing survival included primary tumor site, total bilirubin level, treatment strategies, and remnant tumor burden (all *P* < 0.05). No differences in survival were seen concerning symptoms, number of hepatic lesions, presence of extrahepatic disease, presentation relative to the primary tumor (synchronous vs metachronous), and proliferation rate (Ki-67; Table 3).

**TABLE 3:**
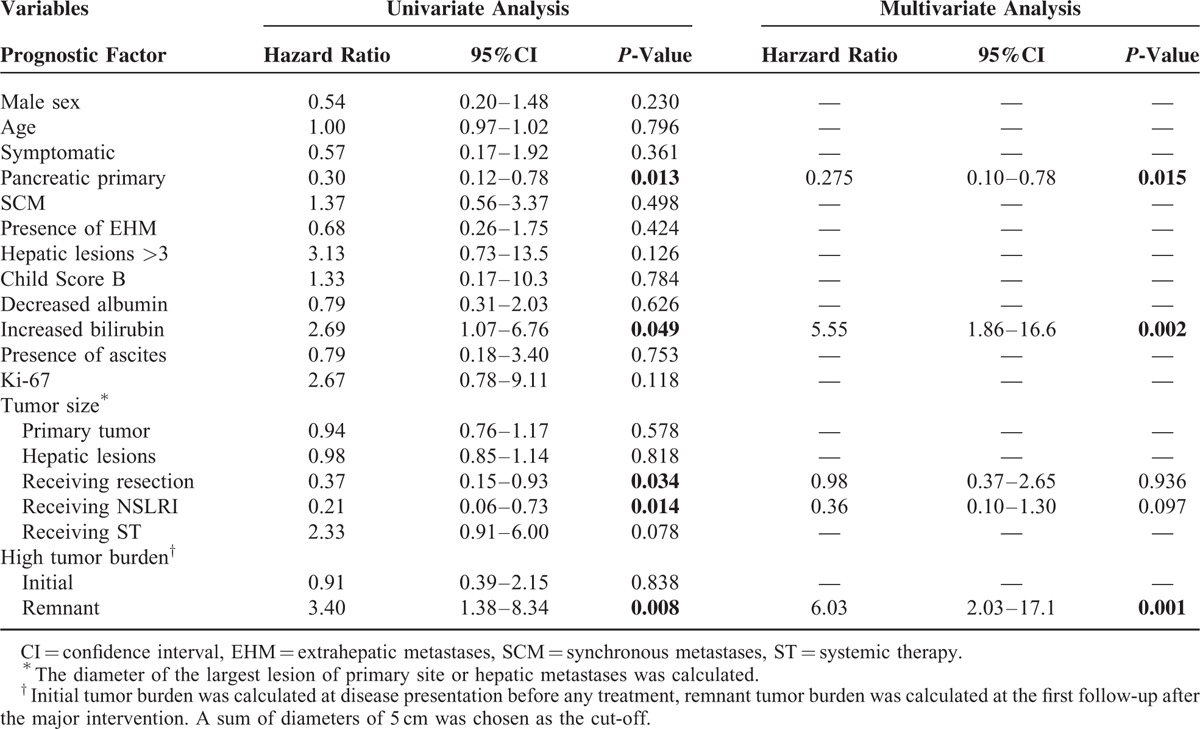
Cox Regression Analyses of Variables Associated With Overall Survival

Patients with pancreatic primary tumors tended to have better prognosis than patients with primary tumors originating from the digestive tract, with a median OS of 54 months (95%CI: 16.1–91.9 months) versus 11 months (95%CI: 0.63–21.4 months; *P* = 0.009). Increased total bilirubin level had a negative impact on patient OS and median OS was 8 months (95%CI: 0.68–15.3 months) versus 27 months (95%CI: 15.2–38.8 months; *P* = 0.028).

Although tumor size of primary or hepatic lesions and initial tumor burden did not correlate with patient survival, remnant tumor burden was shown to strongly relate to patient survival. Patients with a sum of diameters of remnant tumor lesions greater than 5 cm were considered as having “high tumor burden” and those without were regarded as having “low tumor burden.” The median OS of patients in the “low tumor burden” group was 54 months (95%CI: 1.6–106.5 months), while that of patients in the “high tumor burden” group was 15 months (95%CI: 4.5–25.6 months) (*P* = 0.005; Figure 1C).

After controlling for competing risk factors, primary tumor site, total bilirubin level, and remnant tumor burden, were associated with survival (Table 3). Note that “receiving surgery” and “receiving NSLRI” were significant on univariate analyses, but not in the adjusted final multivariate model. This is caused by the strong association between “remnant tumor burden” and the 2 treatment modalities (*P* < 0.001).

## DISCUSSION

The prognosis of GEP G3 NETs is dismal, with the 5-year survival rate varies from 6% to 11%.^5,9^ Most recently, the Surveillance, Epidemiology, and End Results (SEER) program^5^ analyzed data from 2546 patients with GEP G3 NETs and found a median survival of 16 months (95%CI: 15–17 months) for patients with regional disease, and 5 months (95%CI: 4.7–5.4 months) for patients with distant disease. The overall survival in our cohort is longer than these previous studies and the underlying reasons for this discrepancy are complicated. However, in our opinion, the distinct treatment modalities adopted may play an important role, since LT was introduced in 77.8% (n = 28) of patients in our study, ranging from surgery (n = 26) to radiofrequency ablation (n = 3) and intra-arterial therapy (n = 9), separately or combined, with or without ST.

Surgical resection is the mainstay treatment for patients with liver metastases from grade 1 or 2 NETs,^5,10^ and it is recommend to be adopted along with postoperative chemotherapy for GEP G3 NETs with T1/T2N0 disease.^11^ Contrarily, the role of surgical treatment for metastatic GEP G3 NETs has not yet been fully investigated. In an international multiinstitutional cohort^12^ of 339 patients with surgical management of NELM, including 51(15.0%) cases of high grade NELMs and another 111 (32.7%) cases with an unknown grading, liver-directed surgery was demonstrated to prolong patient survival with acceptable tolerance. Similar results^13^ were accumulating, however, as G3 NETs represented only a small fraction of patients, these conclusions may be confounded and needed to be interpreted with caution. And thus, as the first multiinstitutional study specifically confined to patients with liver metastases from GEP G3 NETs, our cohort provides novel and important support of adoption of surgical management for this specific disease population.

Multifocal, bilobular, or even diffuse disseminated hepatic lesions are not uncommon for high grade NELMs (88.3% of cases in our cohort have more than 3 hepatic lesions spreading to 2 lobes).^5,6^ Therefore, anatomic resection of liver metastases often cannot be performed because of insufficient remnant liver volume. To solve this problem, cytoreductive hepatic surgeries with hepatic parenchymal preserving techniques, such as “Cherry Picking,”^14^ were shown to be feasible, safe, and associated with improved survival.^15^ Novel imaging methods^16^ were also developed to accurately predict liver remnant before surgery and to cautiously prevent hepatic failure during perioperative period.

Besides surgery, hepatic ablation and liver-directed intra-arterial therapy are possibly alternative to adjuvant locoregional intervention.^6,10^ Adjuvant ablation,^17^ as well as trans-arterial chemoembolization,^18^ was shown to be safe and provide significant symptom control for patients with metastatic G3 NETs. Moreover, the long-term outcome of patients receiving surgery and liver-directed intra-arterial therapy was found to be almost the same for some asymptomatic NELM patients.^19^ In our study, median OS of patients receiving only surgery and patients receiving only NSLRI was almost equal; however, patients receiving both surgery and NSLRI lived longer, indicating that adjuvant NSLRI may further reduce tumor burden and contribute to long-term disease control in the case of palliative surgery.

Pancreatic primary tumor localization was previously suggested to be a risk factor for decreased survival.^20^ However, conflicting results indicating a favorable prognostic value of pancreatic primary location were reported in recent years.^9,21^ It was hypothesized^9^ that pancreatic G3 NETs tend to have a higher rate of positive somatostatin receptor and a lower Ki67 index, which may contribute to the better prognosis.

Increased bilirubin level had been shown to be a negative prognostic indicator for patients with NELM.^22^ Unlike hepatocellular carcinoma, which often develops in an immunocompromised liver system with chronic virus infection or hepatic cirrhosis, hepatic lesions from NETs usually represent the metastatic potential and invasiveness of the primary tumor, and thus baseline liver function at presentation of the disease may be a possible surrogate of tumor behavior.

Tumor burden is a well-recognized prognostic factor for NELM,^5^ with hepatic tumor involvement <25% being the most commonly mentioned parameter.^19,23^ As 77.8% (n = 28) of patients in our cohort received a type of cytoreductive LT, parameters presenting the initial tumor burden, such as tumor size, sum of diameters, and hepatic involvement at baseline, all failed to correlate with OS, while sum of diameters of the remnant tumor lesions, measured at the first follow-up after major treatment, strongly prognosticated patient outcomes. With the emergence of more sophisticated functional imaging^24^ and morphological evaluation methods,^25^ the relationship between tumor burden, as well as tumor viability, and disease prognosis can be further examined.

Our study also has some limitations. As a retrospective study, the follow-up time interval, imaging modality employed, and data collection process were not unified, and the information was mostly based on medical records, which may lead to certain kind of selection bias. Also, the small sample size could be why we failed to find a significant relationship between ST and outcome of the disease. However, as novel evidence concerning different treatment strategies and prognostic factors of practical use are highly requested, our study provides the very first step to reevaluate the impact of different treatment modalities on G3 GEP NELMs.

In conclusion, aggressive LTs, including surgery, radiofrequency ablation, and liver-directed intra-arterial therapy, are feasible and safe for patients with G3 GEP NELM, and may improve disease outcome. In our opinion, it should be taken into account in designing multidisciplinary treatment plans, as long as the patient has adequate organ function (especially liver function) and is considered well enough to tolerate the surgeries or operations. Future studies are needed to identify candidates who might most benefit from these radical interventions and further studies with larger sample size and prospective design are warranted to reevaluate the current guidelines.
